# Late Blight Resistance Evaluation and Genome-Wide Assessment of Genetic Diversity in Wild and Cultivated Potato Species

**DOI:** 10.3389/fpls.2021.710468

**Published:** 2021-09-30

**Authors:** Yanfeng Duan, Shaoguang Duan, Jianfei Xu, Jiayi Zheng, Jun Hu, Xiaochuan Li, Baoju Li, Guangcun Li, Liping Jin

**Affiliations:** Key Laboratory of Biology and Genetic Improvement of Tuber and Root Crops, Ministry of Agriculture, Institute of Vegetables and Flowers, Chinese Academy of Agricultural Sciences, Beijing, China

**Keywords:** potato, late blight, genetic diversity, microsatellites, single-nucleotide polymorphisms

## Abstract

Late blight, caused by the oomycete *Phytophthora infestans*, is the most devastating disease in potato-producing regions of the world. Cultivation of resistant varieties is the most effective and environmentally friendly way to control potato late blight disease, and identification of germplasms with late blight resistance and clarification their genetic relationship would promote the development of the resistant varieties. In this study, a diverse population of 189 genotypes with potential late blight resistance, consisting of 20 wild species and cultivated *Solanum tuberosum* Andigenum group and Chilotanum group, was screened for the presence of late blight resistance by performing challenge inoculation with four *Phytophthora infestans* isolates including one 13_A2 isolate, CN152. Ten elite resources with broad-spectrum resistance and 127 with isolate-specific resistance against *P. infestans* were identified. To improve the available gene pool for future potato breeding programs, the population was genotyped using 30 simple sequence repeat (SSR) markers covering the entire potato genome. A total of 173 alleles were detected with an average of 5.77 alleles per locus. Structure analysis discriminated the 189 potato genotypes into five populations based on taxonomic classification and genetic origin with some deviations. There was no obvious clustering by country of origin, ploidy level, EBN (endosperm balance number) value, or nuclear clade. Analysis of molecular variance showed 10.08% genetic variation existed among populations. The genetic differentiation (Fst) ranged from 0.0937 to 0.1764, and the nucleotide diversity (π) was 0.2269 across populations with the range from 0.1942 to 0.2489. Further genotyping of 20K SNP array confirmed the classification of SSRs and could uncover the genetic relationships of *Solanum* germplasms. Our results indicate that there exits abundant genetic variation in wild and cultivated potato germplasms, while the cultivated *S. tuberosum* Chilotanum group has lower genetic diversity. The phenotypic and genetic information obtained in this study provide a useful guide for hybrid combination and resistance introgression from wild gene pool into cultivated species for cultivar improvement, as well as for germplasm conservation efforts and resistance gene mining.

## Introduction

Potato (*Solanum tuberosum*) is the most important non-cereal crop plant and ranks third in world food production (Lenman et al., 2016). More than a billion people worldwide consume potato, and global total potato production exceeded 370 million metric tons (FAO, 2019). The prevalent and widespread outbreaks of the devastating late blight (LB) disease, which is caused by the oomycete *Phytophthora infestans*, have long posed a threat to global potato production and food safety (Armstrong et al., 2018). Despite the efforts of breeders and the extensive use of fungicide control measures, there are still concerns regarding the durability and level of resistance against *Phytophthora infestans* (van der Lee et al., 2001). The European LB population, which is derived from new isolates, including the A2 mating type, from Mexico that spread to Europe beginning in 1984, is capable of sexual reproduction and, because of this, may potentially evolve the ability to overcome resistance (*R*) genes in cultivated potato (Goodwin and Drenth, 1997). In the 1990s, the aggressive lineage 13_A2 became widespread in Europe and Asia (Li et al., 2017) and was responsible for severe outbreaks of potato late blight in southwest China. Currently, all 11 *R* genes (*R1-R11*) conferring race-specific resistance derived from *Solanum demissum*, which was extensively used in breeding for LB resistance in potato, have been overcome (Foster et al., 2009). Moreover, some broad-spectrum *R* genes, such as *Rpi-blb1/RB*, *Rpi-sto1*, and *Rpi-pta1,* have also been overcome (Zhu et al., 2010). Therefore, there is a need to identify new resistance germplasms and *R* genes (Foster et al., 2009).

Potato breeders have access to tremendous genetic variation in *Solanum* section *Petota*. There are 107 wild *Solanum* species according to the latest taxonomic treatment (Spooner et al., 2014), distributed in a region ranging from the southwestern United States to Chile, with two centers of diversity, in central Mexico and in the central Andes (Spooner and Salas, 2006). The ploidy of these wild species ranges from diploid to hexaploid (Spooner et al., 2014), and they contain genes encoding numerous traits not found in cultivars; thus, these species represent an especially rich source of disease *R* genes (Cai et al., 2011). Besides *S. demissum*, several other wild *Solanum* species have been reported as being potential sources of LB resistance. These include *Solanum bulbocastanum* (Naess et al., 2000; Park et al., 2005; van der Vossen et al., 2005), *Solanum mochiquense* (Smilde et al., 2005), *Solanum microdontum* (Lokossou et al., 2010), *Solanum pinnatisectum* (Kuhl et al., 2001), *Solanum stoloniferum* (Vleeshouwers et al., 2008; Wang et al., 2008), *Solanum venturii* (Foster et al., 2009), *Solanum americanum* (Witek et al., 2016, 2021), and *Solanum verrucosum* (Chen et al., 2018). Nearly 30 potato LB *R* genes derived from wild *Solanum* species have been cloned (Sun et al., 2016), and some of them, such as *R1*, *R2*, and *R3* and *Rpi-blb1/RB*, have been introgressed into potato cultivars (Park et al., 2005).

In addition to the wild species, there are four cultivated species of *Solanum*, namely, *S. tuberosum*, *S. ajanhuiri* (diploid), *S. juzepczukii* (triploid), and *S. curtilobum* (pentaploid; Ames and Spooner, 2008; Spooner et al., 2014). *S. tuberosum* consists of two cultivar groups, the Andigenum group (diploid, triploid, and tetraploid upland Andean genotypes) and the Chilotanum group (lowland tetraploid Chilean genotypes; Ames and Spooner, 2008; Spooner et al., 2014). Modern cultivars are derived from the Chilotanum group landraces. Cultivated species represent a rich germplasm resource and have tremendous allelic diversity that is of use to potato breeders. The *S. andigenum*, which are classified into the Andigenum group (Spooner et al., 2014), has been found to have developed strong resistance to a wide range of insects and diseases including LB (Sun, 2003). The LB *R* gene *Rpi-phu1* from *S. phureja*, which also classified into the Andigenum group (Spooner et al., 2014), has been identified, and functions in resistance against a wide range of *P. infestans* isolates (Śliwka et al., 2006). Notably, all cultivated *Solanum* species can be readily introgressed into modern cultivars by breeders, and members of both tetraploid *S. tuberosum* cultivar groups can be crossed directly with cultivars (Rodríguez et al., 2010).

Species in *Solanum* section *Petota* have been assigned endosperm balance numbers (EBNs) based on their ability to hybridize with each other (Hanneman, 1994). Matching EBN values between plants are essential for hybridization to produce viable seeds. There exists ploidy and EBN combinations of 6*x* (4EBN), 4*x* (4EBN), 4*x* (2EBN), 2*x* (2EBN), and 2*x* (1EBN) in potatoes (Spooner et al., 2014). Phylogenetic studies in section *Petota* divided the tuber-bearing species into four clades (1–4) based on plastid DNA restriction data (Spooner et al., 1993; Castillo and Spooner, 1997; Rodríguez and Spooner, 1997; Spooner and Castillo, 1997). Subsequent studies using nuclear granule-bound starch synthase I, or waxy and nitrate reductase supported three clades with both results similar to the plastid clades except that the nuclear DNA sequencing data combine species in plastid clades 1+2 (Spooner et al., 2014).

Molecular phylogenetic studies allow estimation of the amount of variation within and between genotypes (Spooner and Nandineni, 2014). Among various molecular markers, simple sequence repeats (SSRs) are very efficient for systematic evaluation of genetic diversity and cultivar identification in highly heterozygous species (Wang et al., 2019) and have been used successfully in potato (Norero et al., 2002; Raker and Spooner, 2002; Braun and Wenzel, 2004; Ghislain et al., 2006, 2009; Duan et al., 2019; Wang et al., 2019). In addition, the SNP array, which has a high density of markers and thus increases the number of genomic loci detected, has been widely used for fingerprinting, diversity analysis, and evolutionary studies in potato (Vos et al., 2015; Ellis et al., 2018). However, to our knowledge, all the studies of genetic diversity to date have been conducted on cultivated potato germplasms. Here, we collected a highly diverse set of 189 potato genotypes including wild *Solanum* species, cultivated *S. tuberosum* Andigenum and Chilotanum group cultivars, with different ploidy levels, EBNs, and clades as determined by nuclear DNA data. Based on these resources, we performed evaluation of late blight resistance with four well-defined *P. infestans* isolates and genome-wide genetic diversity analysis using SSR and SNP markers. The results obtained in this study demonstrated substantial inter- or intra-specific polymorphism and the potential for hybrid combinations to improve heterotic effects.

## Materials and Methods

### Plant Materials

The approximately 3,000 wild and cultivated potato accessions in the Institute of Vegetables and Flowers, Chinese Academy of Agricultural Sciences (IVF, CAAS), China were mainly collected from the Americas, Europe, the International Potato Centre, China; the accessions represent the majority of potato germplasm in China. In this study, 189 genotypes (Table 1; Supplementary Table S1) were selected based on previous LB resistance screening using mixed *P. infestans* isolates (data not shown), pedigree analysis, and field inspection, representing a potential resource for resistant breeding against *P. infestans.* These genotypes covered 20 wild *Solanum* species and both cultivated *S. tuberosum* cultivar groups (the Andigenum group and Chilotanum group) and included all three nuclear clades of section *Petota*. The wild species included 61 genotypes consisting of 35 (57.4%) diploid, 19 (31.1%) tetraploid, and 7 (11.5%) hexaploid accessions, and some of the wild genotypes were derived from Mexico and the Andean regions of Bolivia, Argentina, and Peru. The cultivated *S. tuberosum* Andigenum group included 32 genotypes, of which, six (18.8%; *S. stenotomum*) were diploid and 26 (81.2%; *S. andigenum*) were tetraploid. The wild species and cultivated *S. tuberosum* Andigenum group accessions used in this study are true potato seeds obtained from the United States Potato Genebank (NRSP-6) and the Centre for Genetic Resources, Netherlands (CGN). Each true potato seed, representing a separate genotype, was surface-sterilized, sown *in vitro*. Germinated plants were multiplied and maintained at IVF, CAAS through tissue culture.

**Table 1:** Number of genotypes, countries of origin, ploidy, endosperm balance number (EBN), and nuclear-marker-based clade of *Solanum* spp. evaluated for LB resistance and genetic diversity.

Species	Number of genotypes	Countries of origin^1^	Ploidy	EBN	Nuclear Clade^2^
**Wild species**					
*S. bulbocastanum*	6	GUA, HON, MEX	2*x*	1	1
*S. cardiophyllum*	5	MEX	2*x*	1	1
*S. demissum*	1	GUA, MEX	6*x*	4	Complex
*S. guerreroense*	2	MEX	6*x*	4	Complex
*S. hondelmannii*	1	ARG, BOL, PER	2*x*	2	4
*S. sparsipilum*	2	ARG, BOL, PER	4*x*	4	4
*S. sucrense*	4	ARG, BOL, PER	4*x*	4	4
*S. hougasii*	1	MEX	6*x*	4	Complex
*S. iopetalum*	1	MEX	6*x*	4	3+4
*S. brachycarpum*	1	MEX	6*x*	4	3+4
*S. jamesii*	1	MEX, United States	2*x*	1	1
*S. microdontum*	9	ARG, BOL	2*x*	2	4
*S. mochiquense*	1	PER	2*x*	1	3
*S. oxycarpum*	2	MEX	4*x*	2	3+4
*S. pinnatisectum*	8	MEX	2*x*	1	1
*S. schenckii*	1	MEX	6*x*	4	Complex
*S. polytrichon*	1	MEX, United States	4*x*	2	Complex
*S. stoloniferum*	10	MEX, United States	4*x*	2	Complex
*S. trifidum*	2	MEX	2*x*	1	1
*S. vernei*	2	ARG	2*x*	2	4
**Cultivated species**					
*S. tuberosum* L. Chilotanum group	86		4*x*	4	4
*S. tuberosum* Andigenum group					4
*S. stenotomum*	6		2*x*	2	
*S. andigenum*	26		4*x*	4	
CHS	9		2*x*		
Total	189				

1 *Country code: ARG, Argentina; BOL, Bolivia; CHL, Chile; GUA, Guatemala; HON, Honduras; MEX, Mexico; PER, Peru; URU, Uruguay; and United States, the United States of America. Country of origin cited from* *Spooner et al. (2014)*.

2 *Cladistic relationships are based on nuclear clade investigations as described by* *Spooner et al. (2014)*. *Nuclear clade 1 here includes species placed in both clades 1 and 2 of the plastid results*.

The *S. tuberosum* Chilotanum group consisted of 79 varieties bred from regional breeding programs in China and eight varieties imported from other countries (United States, Netherlands, Poland, Czech Republic, and Canada). An additional set of nine diploid genotypes, which were originally listed as CHS (Canada Hybrid Species) in the IVF, CAAS database, was obtained from Canada; these genotypes were derived from complex *Solanum* hybrids of diploids, which were developed from the diploids induced through prickle pollination from tetraploid *S. tuberosum* with diploid wild species (Table 1). Wild species names follow the taxonomic changes as outlined in Spooner and Salas (2006). The names of the genotypes examined and additional information are available in Supplementary Table S1. *In vitro* grown plants of 189 genotypes were raised in earthen pots (20×25cm^2^) containing a sterile mixture of peat/vermiculite-based compost (1:1, v/v) under the greenhouse conditions at 22°C with a 10h day/14h night photoperiod and a relative humidity of 60–70%.

### Late Blight Resistance Assay

*Phytophthora infestans* Isolates. *P. infestans* isolates used in this study and their virulence profiles are listed in Table 2. These isolates in combination are virulent on plants carrying the *R* genes 1–11. Among these isolates, 80029, T30-4, and 90128 were isolated in Netherlands, and CN152 was isolated in Sichuan Province in China. CN152 is an aggressive 13_A2 isolate, known colloquially as “super-blight” or “blue 13,” which is prevalent throughout the southwest of China and is capable of overcoming the broad-spectrum resistance gene *RB/Rpi-blb1* (Li et al., 2017; Duan et al., 2020).

**Table 2:** The isolates of *Phytophthora infestans* used in this study.

Isolate	Origin	Mating type	Race	Reference
80029	Netherlands	A1	2.4.7	van der Lee et al., 2001
T30-4	Netherlands	A1	1.3.4.5.6.7	Liu et al., 2020
90128	Netherlands	A1	1.3a.3b.4.6.7.8.10.11	Huang et al., 2004
CN152	Sichuan, China	A2	1.3b.4.5.6.7.8.9.10.11	Duan et al., 2020

Detached Leaf Assays. Detached leaf assays were carried out as described by Smilde et al. (2005) with minor modifications. Three leaves per plant for three plants (8 to 10week old) of each genotype were inoculated with two 10-μl droplets of a freshly prepared suspension of zoospores (15,000 sporangia/ml) from 10- to 12-day-old rye agar medium. The inoculated leaves were incubated for 5–7days under 18°C with 16/8h of day and night; 100% humidity was maintained for the first 3days after inoculation, after which a humidity of 70% was restored. The late blight resistance score was determined by visual observation of the spreading lesion size of infected leaves. Scales and ranges of spreading lesions associated with the scale value are as follows: 1 (HR), <3% or no visible infection; 2 (R), 3–10%; 3 (S), 10–30%; 4 (MS), 30–60%; and 5 (HS), >60% (HR=highly resistant; R=resistant; S=sensitive; MS=moderately sensitive; HS=highly sensitive). The susceptible cultivated potato genotype Desiree and the wild species *Solanum jamesii* genotype JAM1-4 with high resistance to 80029, T30-4, 90128, and CN152 (data not shown) were used as negative and positive controls, respectively. Three independent experiments were performed. The Statistical Package for the Social Sciences version 19.0.0 was used for statistical analysis.

### SSR Genotyping

PCR and Electrophoresis. At least three individual plants of each genotype were selected, and young leaves were harvested to obtain high quality DNA. Genomic DNA was extracted according to the modified CTAB procedure of Doyle and Doyle (1987). The template DNA concentration was quantified using a NanoDrop ND-100 and diluted to 25ngμl^−1^ for further analysis. A set of 30 SSR primer pairs covered all 12 potato chromosomes (Supplementary Table S2) with stable and clear amplification, as previously reported (Ghislain et al., 2004; Feingold et al., 2005; Wang et al., 2019), were used to genotype the 189 potato genotypes. SSR PCR amplification was performed in a 10μl reaction, containing 2μl genomic DNA (25ngμl^−1^), 5μl 2×Taq PCR Master Mix (TIANGEN, China), 0.2μl of each primer (10μM), and 2.6μl ddH_2_O. Thermal cycling conditions were 94°C for 5min, 35cycles of 94°C for 30s, primer specific annealing temperature (53–64°C) for 30s, and 72°C for 45s, followed by a final extension of 7min at 72°C. The PCR products were separated on 8% denatured polyacrylamide gel and stained with silver nitrate. Each polymorphic fragment was scored as 1 and 0 for the presence and absence of amplification, respectively.

Structure and Phylogenetic Analysis. The software ADMIXTURE (Alexander et al., 2009) version 1.3.0 was used to analyze the population structure of the 189 potato genotypes by using 173 alleles of 30 SSR markers. For the estimation of the most likely number of genetic groups *K* in a given dataset, a 5-fold cross-validation (CV) approach was applied as implemented in ADMIXTURE. In this approach, each genotype was assigned to one of the populations based on its maximum membership coefficient using a threshold value of 0.60 for the Q statistic according to the optimum *K*-value. Principal Components Analysis (PCA) was employed using GCTA (Yang et al., 2011) version 1.93.2. Matrices were graphically represented using the neighbor-joining (NJ) tree clustering display option of MEGA5 (Tamura et al., 2011) to generate dendrograms for the 189 genotypes and were depicted as trees for phylogenetic analysis.

Genetic Diversity Statistics Analysis. The calculation of percentage of polymorphic loci (p) and polymorphic information content (PIC) was performed as: p=(k/n)×100%, where k is the number of polymorphic loci, and n is the total number of measured loci; $\mathrm{PIC}=1-\overset{i}{\underset{1}{\sum }}f_{i}^{2}$, where *fi* is the frequency of the ith allele in a locus. The software Popgene version 1.32^2^ was used to estimate the *Na* (observed number of alleles), *Ne* (effective number of alleles), *H* (Nei’s genetic diversity), and *I* (Shannon’s information index). Nucleotide diversity (π) was calculated *via* VCFtools (Danecek et al., 2011) version 0.1.16, which was also used to compute the pairwise Fst. Arlequin (Naim et al., 2020) version 3.5.2.2 was used to conduct hierarchical analysis of molecular variance (AMOVA).

### SNP Genotyping

Genotyping with 20K SNP Array. To assess the potential use of SNPs in assessment of genetic relatedness among wild and cultivated potato species, 72 out of the above 189 genotypes were selected for SNP genotyping (Supplementary Table S1). These genotypes represent the range of the *Solanum* species used in our study and the variation in LB resistance. Among the 72 genotypes (28 diploids, 41 tetraploids, and three hexaploids), 26 were wild species, 41 were *S. tuberosum* cultivars (six Andigenum and 35 Chilotanum group cultivars), and five were CHS genotypes. Custom genotyping of 72 genotypes on an Illumina 20K SNP array (GGP Potato V3) was performed according to the manufacturer’s specifications. Genotyping was conducted by Neogen Genomics at the GeneSeek Laboratory in Nebraska, United States. Genotype calls across 21,226 SNPs were generated using GenomeStudio software with default settings. The SNP genotype data were filtered to exclude predetermined low quality SNPs^1^ and any loci with ≥10% missing data. Data quality assessment was done with the default No-Call (GenCall) parameters defined by Illumina, and 50%_GenCall Score and 10%_GenCall Score were generated (Infinium, 2010). Genotypes with a GenCall Score of 0.15 or lower were considered unreliable and designated as no-calls (Infinium, 2010) and omitted from further analysis.

Structure and Phylogenetic Analysis. Population structure was estimated using the program ADMIXTURE (Alexander et al., 2009) by assigning individuals to populations or multiple populations based on SNP genotypes. The most likely number of clusters (*K*) in the dataset was identified using the ADMIXTURE. The program GCTA (Yang et al., 2011) was used to compute PCA on the SNP array genotypes. Software snp2xxx.pl. was used to convert SNP data to MEGA format, phylogenetic tree was constructed by FastTreeMP using the maximum likelihood method, and MEGA5 was applied to decorate the tree.

### Flow Cytometry

Ploidy levels of VRN2-1 and VRN2-5 were determined using a flow cytometer as follows: Approximately 0.5cm^2^ young leaf tissue was chopped with a sharp razor blade in 0.5ml of nuclei extraction buffer (CYStain^™^ UV Precise P, Partec) in a plastic petri dish. The suspension was incubated for 2min at room temperature and then filtered through a 50μM CellTrics^™^ filter into a sample tube. After filtration, 2ml staining buffer (CYStain^™^ UV Precise P, Partec) was added to the isolated nuclei samples. Samples were incubated for 30–60s and then analyzed with a Partec PA flow cytometer.

## Results

### 137 Elite Genotypes With Resistance to Different *Phytophthora Infestans* Isolates Were Identified

**Figure 1:**
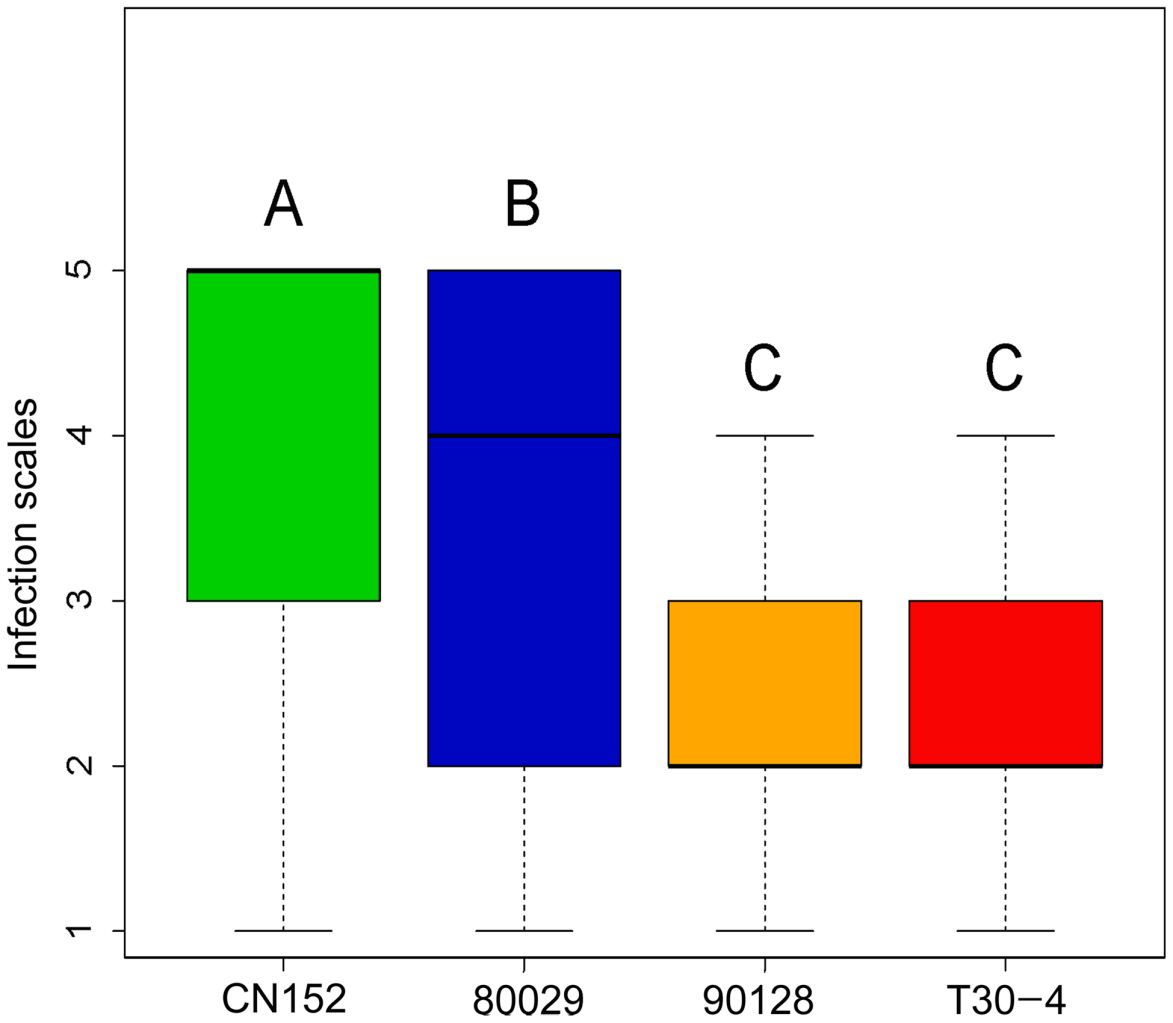
Post-hoc analysis of late blight scores among *Phytophthora infestans* isolates. Bars labeled with a different capital letter at the top of each parameter show a significant difference at *p*<0.01.

Screening of 189 wild and cultivated genotypes for resistance to the *P. infestans* isolates 80029, T30-4, 90128, and CN152, which are of different complexity and exhibit different levels of aggressiveness (Table 2), in detached leaf assays revealed phenotypic variation for resistance (Supplementary Table S1). CN152 (1.3b.4.5.6.7.8.9.10.11) is a 13_A2 isolate that can overcome many of the known late blight *R* genes including the broad-spectrum resistance gene *RB/Rpi-blb1* (Li et al., 2017; Duan et al., 2020). A Tukey’s post-hoc test showed that there were significant differences (*p<*0.01) in pathogenicity among the four *P. infestans* isolates except between 90128 and T30-4; CN152 had the strongest pathogenicity, followed by 80029, and then 90128/T30-4 (Figure 1; Supplementary Table S3).

Of the 189 genotypes tested, 137 were able to resist different *P. infestans* isolates: 10 (5.2%) wild species genotypes originating in Mexico (Table 1) appeared to be resistant to all four *P. infestans* isolates, suggesting that each of these genotypes contains an LB *R* gene(s) other than *R1-R11*; the other 127 genotypes displayed isolate-specific resistance. Of the 10 most resistant genotypes, three (BLB4-29, BLB4-35, and BLB4-38) are *S. bulbocastanum* genotypes, four (CPH1-14, CPH1-21, CPH1-17, and CPH1-6) are *S. cardiophyllum* genotypes, and the other three are *S. jamesii* (JAM1-4), *S. brachycarpum* (BCP1-3), and *S. trifidum* (TRD2-1). *S. brachycarpum* is a hexaploid with an EBN value of 4 and is classified in clade 3+4, and the other four species are all diploids with an EBN value of 1 and are classified in clade 1 (Table 1). The remaining 52 genotypes exhibited susceptibility to all four isolates.

**Figure 2:**
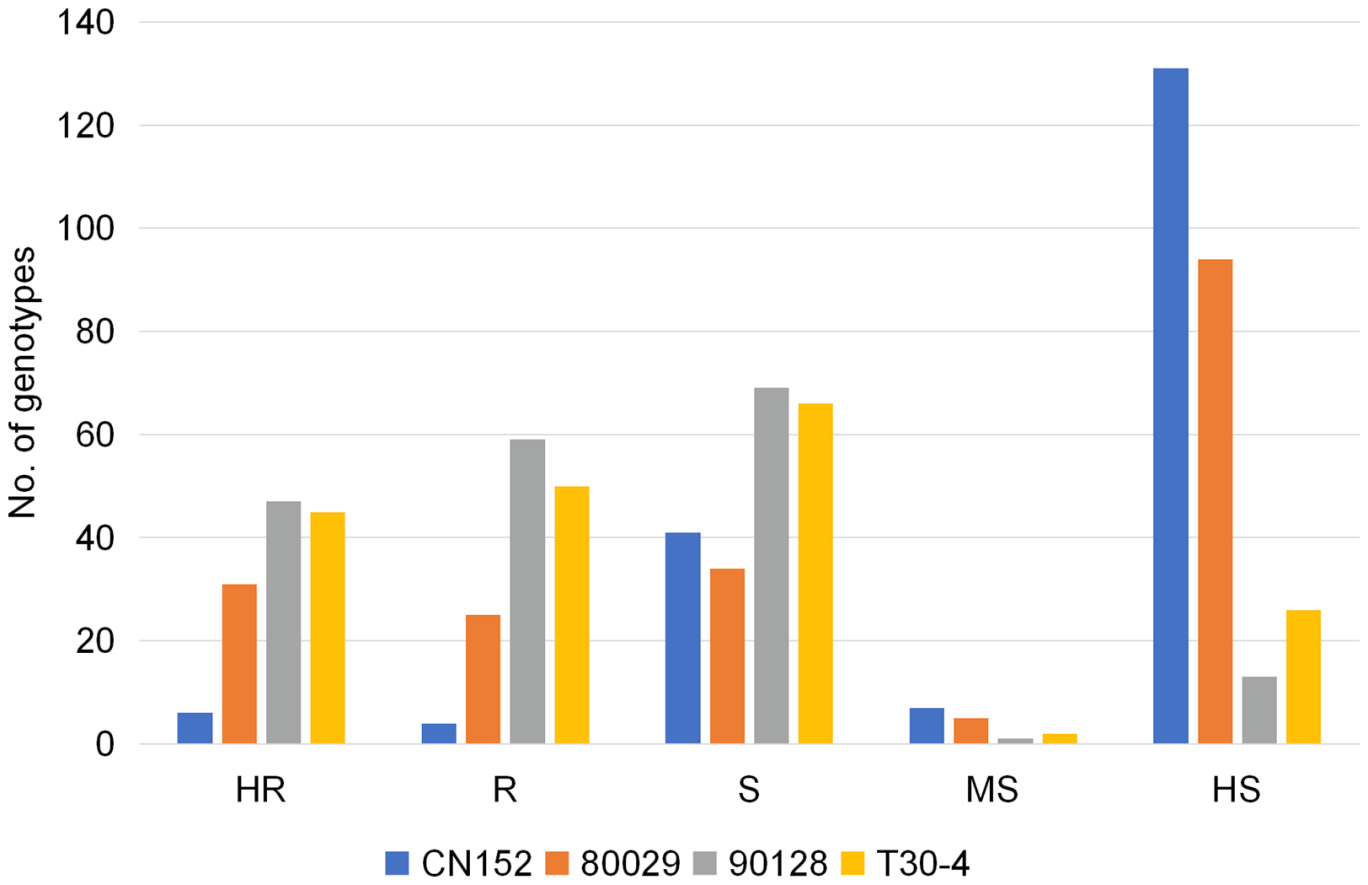
The resistance distribution of 189 potato genotypes in response to four *P. infestans* isolates. Phenotype: highly resistant (HR); resistant (R); sensitive (S); moderately sensitive (MS); and highly sensitive (HS).

Of the 189 genotypes, 106, 95, 56, and 10 showed resistance to isolates 90128, T30-4, 80029, and CN152, respectively, accounting for 56.1, 50.3, 29.6, and 5.3% of all genotypes tested (Figure 2). Among the 137 resistant genotypes, wild species accounted for 43.4, 33.7, 42.9, and 100% of the genotypes that were resistant to 90128, T30-4, 80029, and CN152, respectively, while cultivated species accounted for 54.7, 61.1, 57.1, and 0%, respectively. The highest number of genotypes showed high susceptibility to the 13_A2 isolate CN152 (131, 69.3% of all genotypes tested), followed by 80029 (94, 49.7%) and T30-4 (26, 13.8%). Only 13 genotypes (6.9%) were highly susceptible to isolate 90128, indicating that there were differences in aggressiveness among the four isolates and 90128 is the least aggressive. The raw data from the screening are available in Supplementary Table S1.

### SSR Genotyping Revealed Abundant Genetic Diversity and Distinctiveness of 189 Potato Genotypes

Thirty SSR markers distributed over all 12 potato chromosomes were used to genotype the entire population of 189 genotypes (Supplementary Table S2). A total of 171 polymorphic alleles was detected (Supplementary Table S4). A high richness of alleles was observed with an average of 5.77 alleles per primer pair, ranging from 3 to 12 alleles per primer pair. The PIC values for the markers ranged from 0.1975 to 0.5329 with an average of 0.3559. The effective alleles per locus (Ne) ranged from 1.0839 to 1.6674 while Nei’s gene diversity (H) ranged from 0.0715 to 0.3812, and Shannon’s information index (I) ranged from 0.1428 to 0.5670 (Table 3).

**Table 3:** Genetic diversity parameters of 30 SSR markers evaluated in 189 potato genotypes.

Marker	Na^*^	Ne^*^	H^*^	I^*^	Total alleles	Polymorphic alleles	% Polymorphic alleles	PIC^*^
STM1049	2	1.3794	0.2499	0.3959	4	4	100.0	0.3011
STM2022	2	1.4132	0.2531	0.3954	6	6	100.0	0.3414
STM1053	2	1.2557	0.1869	0.3195	5	4	80.0	0.3410
STPoAc58	2	1.5526	0.3140	0.4593	4	4	100.0	0.4108
STM0019a	2	1.3331	0.2111	0.3395	8	8	100.0	0.4119
STM2023	2	1.6372	0.3812	0.5670	5	5	100.0	0.5329
STM1104	2	1.3251	0.2218	0.3693	4	4	100.0	0.3674
STM3012	2	1.3546	0.2052	0.3195	5	5	100.0	0.1975
STM1106	2	1.3528	0.2031	0.3231	7	7	100.0	0.3507
STM0037	2	1.4358	0.2455	0.3747	7	7	100.0	0.3539
S118	2	1.5690	0.3302	0.4993	5	5	100.0	0.4190
S180	2	1.4383	0.2733	0.4259	8	8	100.0	0.3488
S25	2	1.5101	0.3128	0.4812	4	4	100.0	0.3336
S7	2	1.6251	0.3755	0.5603	6	6	100.0	0.3739
S151	2	1.2667	0.1739	0.2877	12	12	100.0	0.3141
S184	2	1.5680	0.3300	0.4934	5	5	100.0	0.3835
S192	2	1.4784	0.2745	0.4100	5	5	100.0	0.3535
S170	2	1.6674	0.3635	0.5296	5	5	100.0	0.3903
S174	2	1.3999	0.2440	0.3848	6	6	100.0	0.4206
S122	2	1.0839	0.0715	0.1428	5	5	100.0	0.2604
S168	2	1.2357	0.1807	0.3141	10	10	100.0	0.3337
S120	2	1.2902	0.2116	0.3577	6	5	83.3	0.3718
S148	2	1.6162	0.3391	0.4913	4	4	100.0	0.4319
S153	2	1.5679	0.3301	0.4995	4	4	100.0	0.4123
S162	2	1.2052	0.1691	0.3089	3	3	100.0	0.3003
S182	2	1.4142	0.2484	0.3796	4	4	100.0	0.3052
S183	2	1.3899	0.2540	0.4075	3	3	100.0	0.2515
S187	2	1.3542	0.1995	0.3022	9	9	100.0	0.2399
S188	2	1.3830	0.2635	0.4188	7	7	100.0	0.4472
S189	2	1.4879	0.3153	0.4900	7	7	100.0	0.3768

^*^Na, observed number of alleles; ^*^Ne, effective number of alleles; ^*^H, Nei’s gene diversity; ^*^I, Shannon’s information index; and ^*^PIC, polymorphic information contents.

**Figure 3:**
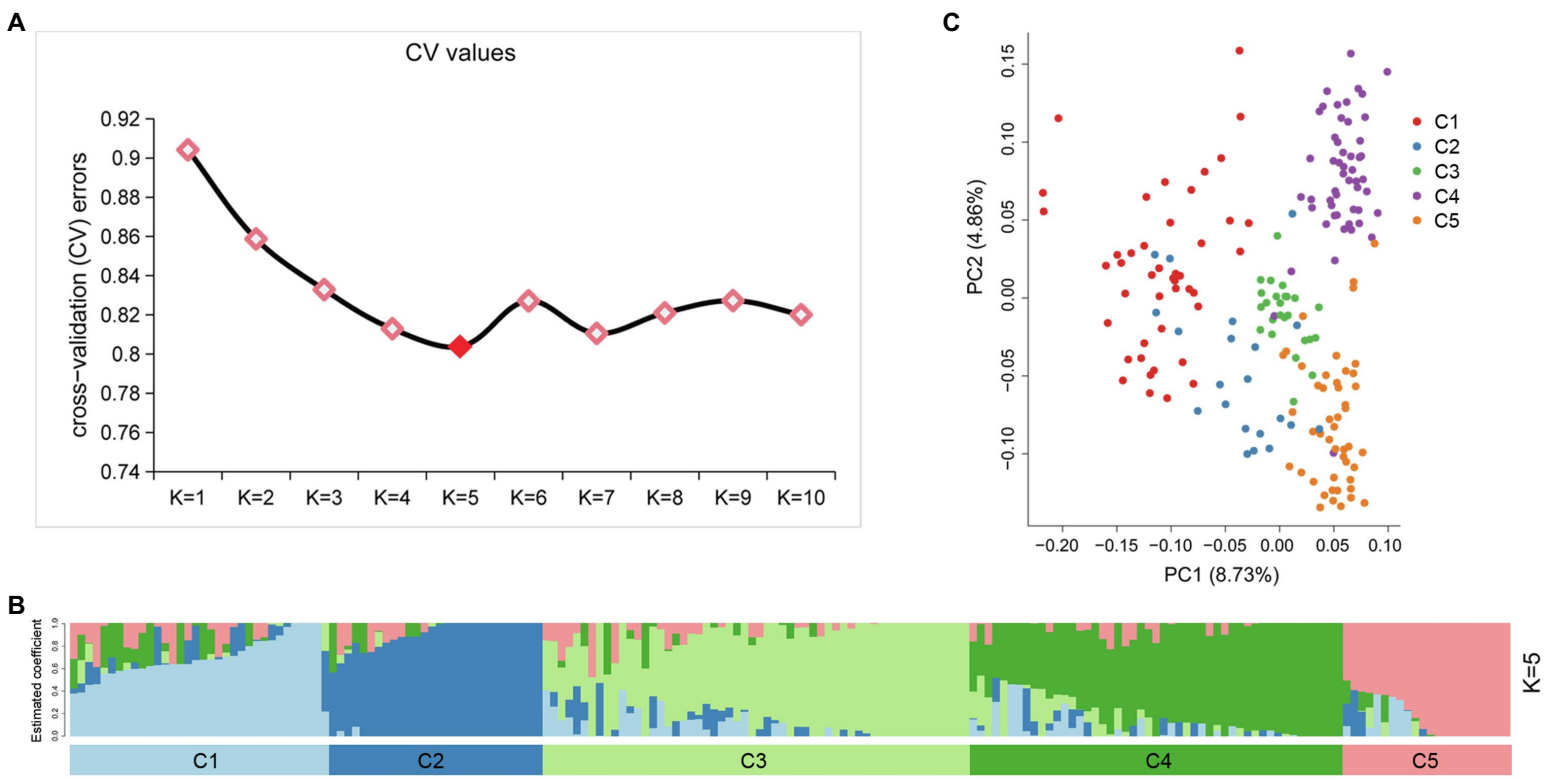
Population structure of 189 potato genotypes based on SSR markers, the graphical presentation of estimation of **(A)** the cross-validation error plot exhibited as implemented in ADMIXTURE. **(B)** Population structure inferred using ADMIXTURE with *K*-value at five. **(C)** Principal component analysis using GCTA software.

An admixture model-based approach was implemented to investigate the population structure of 189 potato genotypes. The optimum cluster was five, which was generated from ADMIXTURE with the lowest cross-validation (CV) error values (Figure 3A). The 189 genotypes were classified into five populations based on their maximum membership coefficients, which were designated as C1 to C5 (Figure 3B). PCA was also used to evaluate the data structure and underlying variance. As in the structure analysis, five populations were distinguished by the PCA plot with some admixture (Figure 3C). The first and second principal coordinates explained 8.73 and 4.86% of the genetic variation, respectively. The five population could group into three clusters: C1, C2 each represented a cluster, while C3, C4, and C5 represented one cluster.

**Figure 4:**
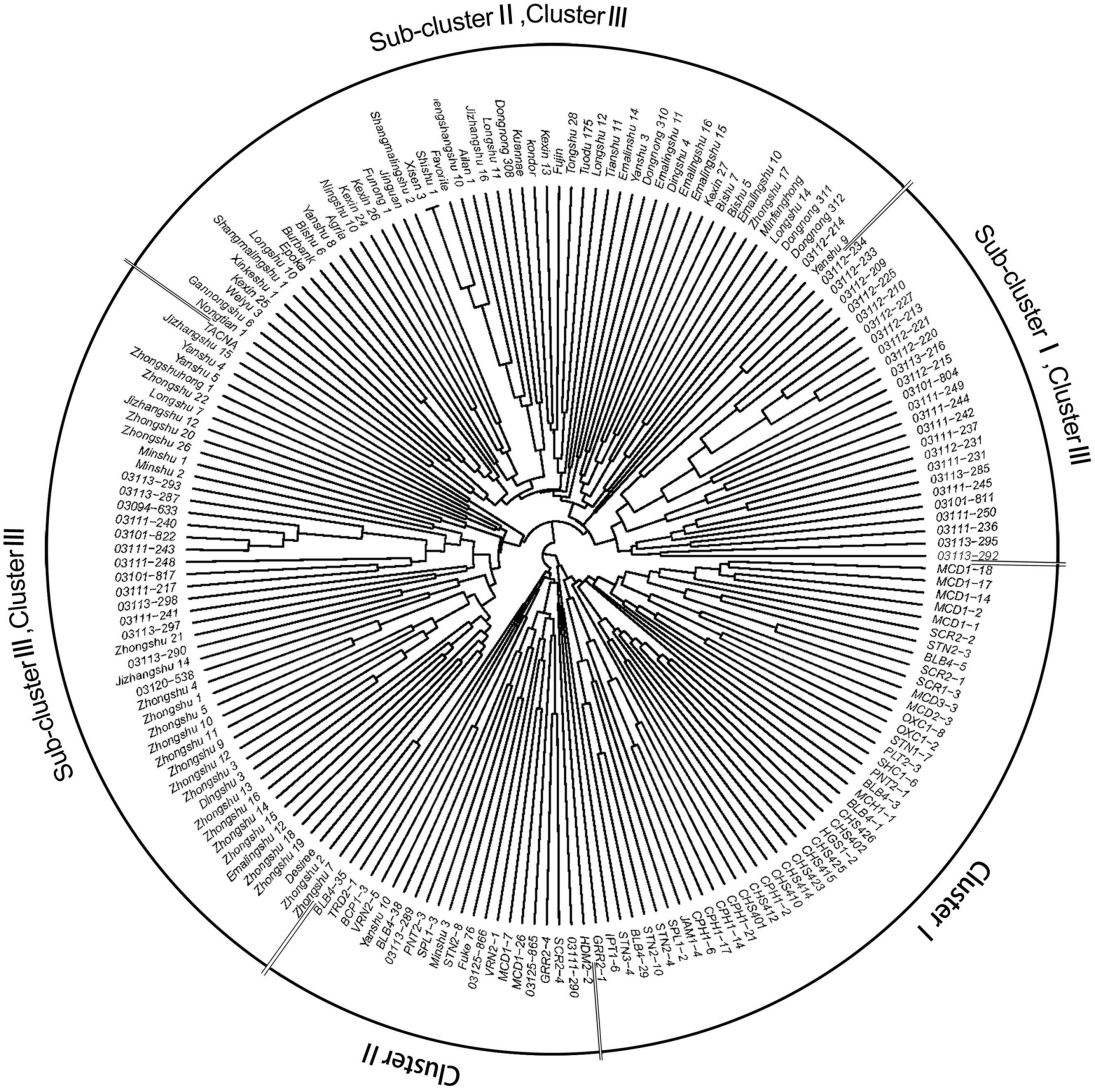
Neighbor-joining tree based on a dissimilarity matrix of 189 potato genotypes examined with 30 SSR markers.

NJ phylogenetic analysis was used to detect the genetic relationship across 189 genotypes based on the dissimilarity matrix (Figure 4). Three main clusters were observed as: clusters I, II, and III with 44, 21, and 124 genotypes, respectively. Cluster I, corresponding to C1 in population structure, included 35 wild species genotypes and all nine CHS genotypes. Cluster II (C2) included 18 wild species genotypes and four *S. tuberosum* genotypes. Cluster III could be further subdivided into three sub-clusters: sub-clusters I, II, and III with 25, 52, and 47 genotypes, respectively. Sub-cluster I (C3) formed a distinct cluster with *S. tuberosum* Andigenum group genotypes, with exception of six wild species genotypes. Sub-cluster II (C4) contained *S. tuberosum* Chilotanum group genotypes, with exception of one Andigenum group genotype. Sub-cluster III (C5) mainly included genotypes of the *S. tuberosum* Chilotanum group, with a few exceptions. The cluster results showed a clear separation of wild species and *S. tuberosum* germplasm. Furthermore, the *S. tuberosum* Andigenum and Chilotanum germplasm differentiated into different groups. There was no obvious clustering by country of origin, ploidy level, EBN value, or clade based on nuclear DNA data. In most cases, the clusters in the dendrogram fit well with the recorded pedigree information; for example, Chilotanum group cultivars Kexin 13, Zhengshangshu 10, Shishu 1, Xisen 3, Jinguan, and Funong 1, which share the Favorita pedigree, all clustered together with Favorita. In addition, tetraploid genotypes of the wild species *S. stoloniferum* were distributed among all clusters, suggesting that there is considerable gene flow between wild and cultivated species.

Both AMOVA and pairwise Fst analysis were performed to investigate the genetic variations among the five populations. The results showed that 10.08% of the total genetic variation occurred among populations (Table 4). Genetic differentiation was indicated by pairwise Fst ranging from 0.0937 to 0.1764 (Table 5). The highest level appeared between cluster I and sub-cluster II (cluster III), whereas the lowest appeared between cluster I and cluster II. Furthermore, nucleotide diversity (π) and Shannon’s diversity index of the populations were calculated. The π was 0.2269 across populations, 0.1942 in sub-cluster I (cluster III) and 0.2489 in cluster I, and Shannon’s diversity index ranged from 0.3004 (sub-cluster I, cluster III) to 0.3965 (cluster I; Table 6).

**Table 4:** Analysis of molecular variance based on SSRs for populations of 189 genotypes.

Source of variation	*d.f.*	Sum of squares	Variance components	Percentage variation
Among populations	4	126.4330	0.4675	10.08%^***^
Among individuals within populations	182	1478.9840	4.1722	89.92%^***^
Within individuals	187	0	0	0^***^
Total	373	1605.4170	4.6397	100.00%

d.f. degrees of freedom; ^***^significant of data rand probability; and p<0.001.

**Table 5:** The matrix of pairwise genetic differentiation among populations (Fst) based on SSRs.

Population	Cluster I	Cluster II	Cluster III-Sub I	Cluster III-Sub II	Cluster III-Sub III
Cluster I	0				
Cluster II	0.0937	0			
Cluster III-Sub I	0.1473	0.1373	0		
Cluster III-Sub II	0.1764	0.1520	0.1463	0	
Cluster III-Sub III	0.1706	0.1213	0.1403	0.1024	0

**Table 6:** Diversity statistics of the populations across *Solanum* germplasm based on SSRs.

Population	Sample size	Nucleotide diversity (π) value^*^	Shannon’s index^*^
Cluster I	44	0.2489	0.3965
Cluster II	21	0.2407	0.3827
Cluster III-Sub I	25	0.1942	0.3004
Cluster III-Sub II	52	0.2278	0.3573
Cluster III-Sub III	47	0.2227	0.3530

* *Nucleotide diversity (π) value was calculated via VCFtools and Shannon’s index was estimated using Popgene software*.

The results showed that the 10 most resistant wild species genotypes (BLB4-29, BLB4-35, BLB4-38, CPH1-6, CPH1-17, CPH1-14, CPH1-21, JAM1-4, BCP1-3, and TRD2-1) were distributed into the two wild species clusters, cluster I and II (Figure 4), and the 52 genotypes which were susceptible to all four *P. infestans* isolates were distributed into all clusters and sub-clusters, with eight, four, 14, 10, and 16 in cluster I, cluster II, sub-clusters I, II, and III, respectively. There seems no discernable relationship between the distribution of resistant phenotypes and SSR genotyping.

### The 20 K SNP Array Proved Useful in Confirming Identity and Highlighting the Diversity in the Subset of 71 Genotypes

To further verify the results of classification using SSRs and to assess the utilization of SNPs in establishing genetic relationships, 72 out of 189 genotypes were selected for SNP genotyping using the Infinium 20KV3 array. The average call rate of the 72 genotypes (percentage of the 21,226 SNPs called) was 97.24% (Supplementary Table S5). Due to the lowest call rate (38.75%) and 10%_GenCall Score (0.0766), the *S. pinnatisectum* genotype 03101-817 was dropped from further study. Among the 33 genotypes with call rates higher than 99%, 31 were *S. tuberosum* Chilotanum group genotypes and two were wild species genotypes. Genotyping data of 72 genotypes on the 20K SNP array are available in supplementary file.

**Figure 5:**
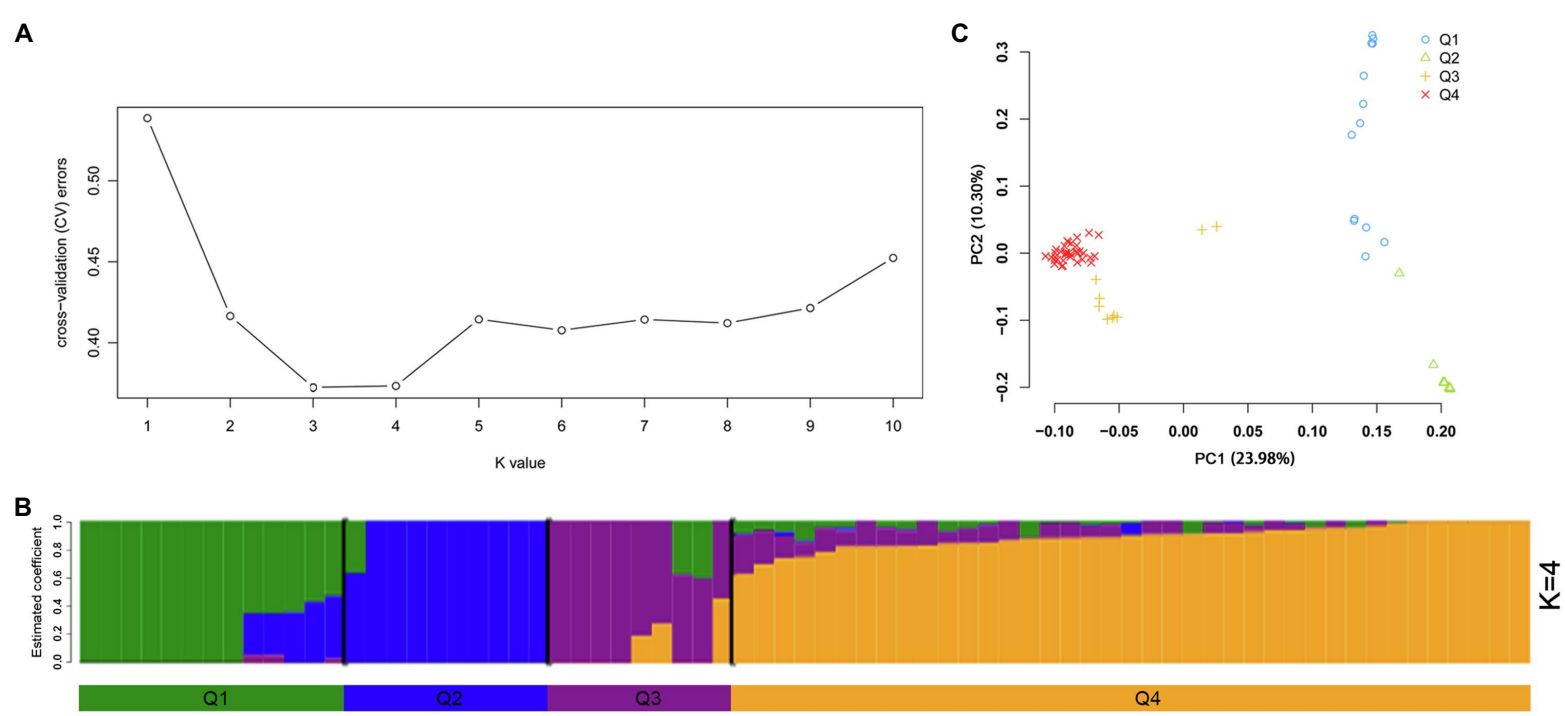
Population structure of 71 potato genotypes based on SNP array, the graphical presentation of estimation of **(A)** the cross-validation error plot exhibited as implemented in ADMIXTURE. **(B)** Population structure inferred using ADMIXTURE with *K*-value at four. **(C)** Principal component analysis using GCTA software.

**Figure 6:**
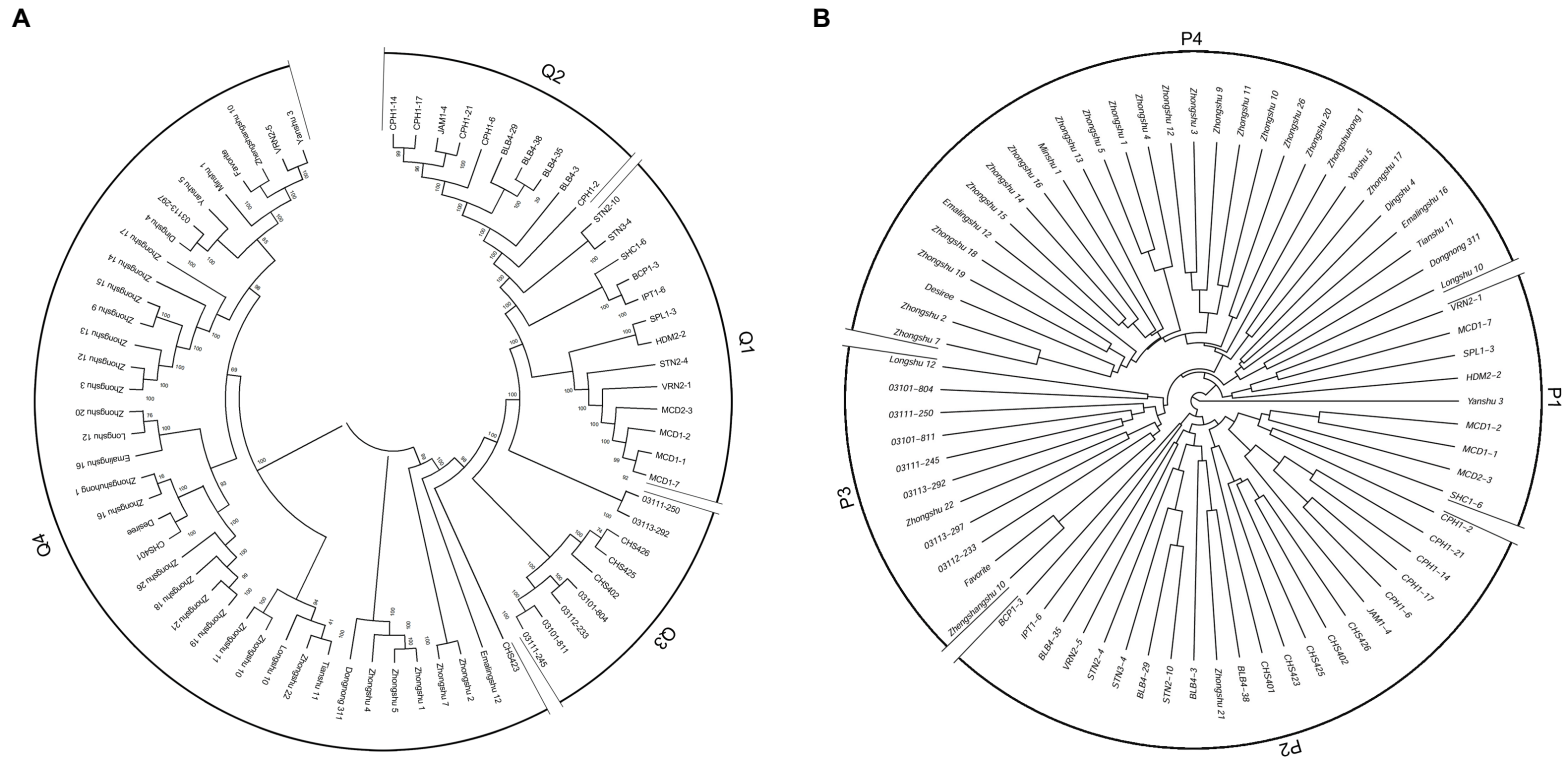
Phylogenetic tree of 71 potato genotypes based on: **(A)** SNP array data. **(B)** SSR data with 30 markers.

The program ADMIXTURE was employed to evaluate admixture and the number of populations in this dataset. Strong support was observed for *K* =4 (Figure 5A). At *K* =4, four populations were apparent with minor admixture within these groups (Figure 5B). Similar to the admixture analysis, PCA also demonstrated four populations within the 71 potato genotypes (Figure 5C; Supplementary Table S6). The first two principal coordinates explained 34.28% of the genetic variation across the potato genotypes. A phylogenetic tree was constructed using the maximum likelihood method (Figure 6A). The four clusters were composed predominantly of Q1 wild species (13 genotypes covering five wild species), Q2 wild species of *S. bulbocastanum*, *S. cardiophyllum*, and *S. jamesii* (10 genotypes), Q3 a mixed collection of three wild species genotypes, three *S. tuberosum* Andigenum group genotypes, and three CHS genotypes, and Q4 *S. tuberosum* Chilotanum group germplasm (35 genotypes), with some exceptions. The results above demonstrated that SNPs can clearly separate wild species from *S. tuberosum* germplasms. It was interesting that the five CHS genotypes, which are the progenies of wild species and *S. tuberosum*, differentiated into two groups. In addition, the tetraploid *S. stoloniferum* genotype 03113-297 grouped together with the *S. tuberosum* Chilotanum group. *S. tuberosum* Chilotanum group genotypes Zhongshu 9, Zhongshu 13, Zhongshu 14, and Zhongshu 15 are all derived from Shepody and Zhongshu 3, and these four genotypes grouped together.

Compared with LB resistance, it was found that *S. bulbocastanum* genotypes (BLB4-29, BLB4-35, and BLB4-38) and *S. cardiophyllum* genotypes (CPH1-14, CPH1-21, CPH1-17, and CPH1-6) that showed the highest level of LB resistance were all grouped into Q2. Although these two diploid Mexican wild potato species are considered to have a sister group relationship in taxonomy based on chloroplast analysis (Rodríguez and Spooner, 1997), the two susceptible genotypes (BLB4-3 and CPH1-2) in this cluster were grouped separately from the resistant genotypes. The 23 genotypes with susceptible to all four *P. infestans* isolates were distributed in Q1, Q3, and Q4, while the genotypes showing similar resistance level were clustered together, such as Zhongshu 3 and Zhongshu 12 with isolate (90128)-specific resistances were clustered together, Zhongshu 2 and Zhongshu 7 showing high susceptibility to all four isolates were clustered together in Q4.

To compare the SSR and SNP marker systems, 30 SSRs were analyzed for a subset (71) of the 189 genotypes. A phylogenetic tree was produced, and the 71 genotypes could also be divided into four clusters: P1 included eight wild species genotypes, with one exception, Yanshu 3, which is *S. tuberosum* Chilotanum group cultivar (compared to SNP results, all the wild species genotypes in P1 classified in Q1); P2 was a mixed group containing 13 wild, five CHS, three *S. tuberosum* Andigenum, and one Chilotanum group genotype, respectively (P2 included all the 10 genotypes in Q2); P3 included four wild species genotypes and seven *S. tuberosum* germplasms (P3 included all the genotypes in Q3, with the exception of CHS genotypes); and P4 included 29 genotypes, which are all *S. tuberosum* Chilotanum group cultivars (all the 29 genotypes in P4 classified in Q4; Figure 6B).

## Discussion

### LB Resistance Evaluation of Different Germplasm Resources Provides Useful Information for Potato Resistance Breeding and *R* Gene Mining

Potato breeders have access to diverse germplasms with tremendous genetic variation. The potato research group at IVF, CAAS maintains a collection of 3,000 potato accessions, from which breeders can choose germplasm for resistance evaluation. In a previous study, we conducted preliminary screening of the majority of these germplasms using mixed *P. infestans* isolates (data not shown). Combined with pedigree analysis and field investigation, 189 genotypes with potential LB resistance were selected for further evaluation using four individual *P. infestans* isolates. These genotypes represent 20 wild *Solanum* species and both cultivated *S. tuberosum* Andigenum group and Chilotanum group germplasms. Of the 189 genotypes tested, 134 (69.8%) appeared to be highly susceptible to the 13_A2 isolate CN152 (Li et al., 2017; Duan et al., 2020), confirming the strong aggressiveness of CN152. The 88 tetraploid varieties bred from regional breeding programs in China or imported from other countries were all overcome by CN152, indicating the importance of LB resistance evaluation and the significance of introduction of strong genetic resistance from wild species into cultivated species. We observed that there exists large resistance variation among genotypes within species. For example, *S. bulbocastanum* genotype BLB4-1 exhibited susceptibility to all four *P. infestans* isolates, while BLB4-29, BLB4-35, and BLB4-38 appeared to be resistant to all four isolates, implying that it is important to focus on genotypes rather than species.

In total, there was 127 genotypes identified as resources with isolate-specific resistance. Among them, the *S. andigenum* genotype 03112–233 and *S. tuberosum* L. genotype Longshu 12 were both resistant to 90128 and T30-4 but susceptible to CN152 and 80029 (Supplementary Table S1). Previous diagnostic resistance gene enrichment sequencing (dRenSeq) analysis revealed that there are no known nucleotide-binding and leucine-rich repeat genes in 03112-233 (Duan et al., 2020) or Longshu 12 (Duan et al., 2021). The dRenSeq results suggest that one or more other, so far uncharacterized *R* genes, underpin the observed resistance to isolates 90128 and T30-4. Although resistance has been overcome by the isolates CN152 and 80029, 03112–233 and Longshu 12 may still be of significant practical value in combatting LB in locations where *P. infestans* populations are known to contain no new races that evade recognition by the resistance gene. The germplasms with isolate-specific resistance that we identified in our study can be applied for LB-resistant breeding based on population structure analysis of *P. infestans* in different potato production areas.

Ten genotypes with high resistance to all four *P. infestans* isolates were identified in the present study, all of which originated in Mexico, and are among the five wild species *S. bulbocastanum*, *S. cardiophyllum*, *S. jamesii*, *S. brachycarpum*, and *S. trifidum*. This finding further confirms the strong resistance to LB in wild species derived from Mexico, which is presumably a consequence of close co-evolution with the pathogen *P. infestans* (Hein et al., 2009). Of the 10 LB-resistant genotypes, BLB4-29, BLB4-35, and BLB4-38 are diploid *S. bulbocastanum* genotypes. Several *R* genes have been cloned from *S. bulbocastanum*, including R*pi-blb1/RB*, *Rpi-blb2*, and *Rpi-blb3*, and all of them confer broad-spectrum resistance to LB (Song et al., 2003; van der Vossen et al., 2003; Park et al., 2005; van der Vossen et al., 2005). CPH1-6, CPH1-14, CPH1-17, and CPH1-21 are diploid *S. cardiophyllum* genotypes. Chloroplast DNA study supported the designation of *S. bulbocastanum* and *S. cardiophyllum* as sister taxa (Rodríguez and Spooner, 1997). Indeed, an allele mining strategy in *S. cardiophyllum* previously identified functional orthologs of *Rpi-blb1/RB* (Lokossou et al., 2010). JAM1-4 is a diploid *S. jamesii* genotype, and previous dRenSeq analysis revealed that JAM1-4 may contain a novel *R* gene(s) (Zheng et al., 2020). BCP1-3 and TRD2-1 are hexaploid *S. brachycarpum* and diploid *S. trifidum* genotypes, respectively, and no cloned *R* gene has been reported in these two species. The 10 genotypes may provide a very useful source for potential *R* gene(s) mining as well as LB resistance breeding.

One potential issue with using wild genotypes as a source of *R* genes is the crossing barriers that exist between cultivated potato and most wild species. However, these barriers, which are the consequence of differences in EBN, can be easily overcome using ploidy manipulations and bridge crosses (Jansky, 2006). Introduction of *S. bulbocastanum*-derived resistance has been achieved through interspecific bridge crosses between *S. acaule*, *S. bulbocastanum*, *S. phureja*, and *S. tuberosum* (Hermsen and Ramanna, 1973), resulting in so-called ABPT material that is widely used for potato LB breeding. Additional methods to produce fertile interspecific hybrids include mentor pollination, embryo rescue, hormone treatments, reciprocal crosses, selection of cross-compatible genotypes, and somatic fusion (Jansky, 2006). For example, Chandel et al. (2015) generated somatic hybrids between *S. cardiophyllum* and cultivated potato, and these interspecific somatic hybrids gave rise to fertile plants that retained resistance and could be used for breeding.

### Assessment of Genetic Diversity Among Diverse Potato Germplasm Resources Provides the Theoretical Foundation for Parent Selection of Potato Breeding and Germplasm Conservation Efforts

LB disease contributes to extensive crop losses in China; in particular, the clonal lineage 13_A2 has become prevalent throughout the southwest of China. In recent decades, numerous crop improvement programs in China have resulted in the development of more than 600 potato varieties (Duan et al., 2019); however, few can resist the aggressive 13_A2 *P. infestans* isolate. Furthermore, due to the large contribution of several elite parents to breeding (Li et al., 2018), the genetic base of potato varieties in China is narrow (Duan et al., 2019). The challenge for breeders remains that of broadening the genetic base and combining high levels of late blight resistance with yield, early maturity, and quality. In the present study, we obtained 137 genotypes with LB resistance; these included 10 wild genotypes that can resist the 13_A2 isolate CN152, one of the most aggressive isolates in China. We also used SSR markers to assess the genetic diversity of these potato germplasms to bridge the gap between the potato geneticists and breeders in cultivar improvement programs. Our results revealed that there exits abundant genetic variation in the germplasms of this study with a high Nei’s gene diversity value 0.2577 (Table 3). The wild species groups have the highest level of genetic diversity with Shannon’s information index of 0.3965 and 0.3827 for cluster I and cluster II, respectively, while the cultivated *S. tuberosum* Andigenum group and Chilotanum groups have lower genetic diversity with Shannon’s index range from 0.3004 to 0.3573. Our results provide valuable information for parent selection of potato breeding and resistance introgression from wild species into cultivated *S. tuberosum* groups.

Simple sequence repeat markers are preferred for genome-wide studies of genetic variation because of their high clarity and reproducibility, low cost, and suitability for multiplexing use with low quality DNA. The 30 SSR markers used in the present study were selected from among the highly informative and user-friendly markers developed for genotyping cultivated potato (Ghislain et al., 2004; Feingold et al., 2005; Duan et al., 2019; Wang et al., 2019). However, our study included not only cultivated potato species, but also 20 wild species with different ploidy levels, EBNs, and clades as determined by nuclear DNA data. To further verify the results of classification using SSRs and to assess their potential use in assessment of genome-wide variation for diversity studies in potato, 72 out of 189 genotypes were selected for SNP genotyping (21,226 SNPs). There was considerable concordance between the SSR and SNP results, and this was evident from the fact that both SSRs and SNPs could: (1) separate wild species and *S. tuberosum* germplasm and differentiate between different wild species, and (2) group the genotypes with close genetic relationships together; for example, Zhengshangshu 10, which is an advanced mutant line of Favorita, grouped together with Favorita in both SSR and SNP trees. However, there were also some inconsistencies between the two results; for example, *S. cardiophyllum* and *S. bulbocastanum*, the sister taxa grouped together directly based on the results of SNPs but not SSRs; and the four tetraploid varieties of Zhongshu 9, Zhongshu 13, Zhongshu 14, and Zhongshu 15, the progeny of Shepody × Zhongshu 3, were also clustered together based on SNPs, while not based on SSR markers. The differing SSR and SNP results may be related to the fact that the 20K SNP array was developed with a high density of markers (on average, one SNP per 40 kbp) and allowed us the opportunity to broaden the number of genomic loci detected. The SNP array has been widely used for fingerprinting and diversity analysis and evolutionary studies in potato (Vos et al., 2015; Ellis et al., 2018). However, in laboratories with limited resources and instrumentation facilities, SSR marker systems provide an easy, reliable, and cost-effective tool for scanning the whole genome for genetic and genomic studies.

Neutral genetic markers are also commonly used to characterize germplasm collections and in conservation studies, with the goal of improving the value of these germplasms to breeders (Brow, 1989). The *S. venturii* genotypes were originally listed as *S. okadae* in the CGN database but have been reclassified based on work using amplified fragment length polymorphism markers (Jacobs et al., 2008). In the present study, *S. jamesii* genotype JAM1-4 was clustered between the *S. cardiophyllum* genotypes in dendrograms based on SNPs (Figure 6A), and also together with *S. cardiophyllum* in those based on SSR results (Figure 6B), indicating that the genotype JAM1-4 could be reclassified as *S. cardiophyllum*. This classification needs to be further investigated using morphological traits and genomic information. Additionally, VRN2-5, which is described as diploid *S. vernei* in the IVF, CAAS database, was clustered with other genotypes of the Chilotanum group. Morphological inspection found that VRN2-5 has much more vigorous growth and larger size of leaves than that of the *S. vernei* genotypes VRN2-1. Flow cytometric analysis showed that VRN2-5 is tetraploid, while VRN2-1 is diploid, which further confirmed that VRN2-5 was mislabeled and should be considered a member of the Chilotanum group.

Molecular genetic information about the extent of diversity and the intra- and inter-relationships among different potato germplasms possessing desirable agronomic traits would greatly benefit plant breeding programs aimed at generating new high-yielding and disease-resistant varieties. To our knowledge, this is the first report of comparative assessment of genetic diversity among potato germplasms using SSR and SNP marker systems, and our findings offer a useful guide for crop improvement and germplasm conservation programs in potato.

## Data Availability Statement

The original contributions presented in the study are included in the article/Supplementary Material, and further inquiries can be directed to the corresponding authors.

## Author Contributions

YD conducted the experiments, performed the data analysis, and wrote the manuscript. SD and JX helped in material management and data analysis. JZ and JH participated in the experiments and data analysis. XL analyzed the data and wrote the manuscript. BL designed the study. GL and LJ designed the study and reviewed and edited the manuscript. All authors contributed to the article and approved the submitted version.

## Funding

This research was funded by the Agricultural Breeding Project of Ningxia Hui Autonomous Region, China, grant number 2019NYYZ01-1, China Agriculture Research System, grant number CARS-9, and the National Natural Science Foundation of China, grant number 31561143006.

## Conflict of Interest

The authors declare that the research was conducted in the absence of any commercial or financial relationships that could be construed as a potential conflict of interest.

## Publisher’s Note

All claims expressed in this article are solely those of the authors and do not necessarily represent those of their affiliated organizations, or those of the publisher, the editors and the reviewers. Any product that may be evaluated in this article, or claim that may be made by its manufacturer, is not guaranteed or endorsed by the publisher.

## Footnotes

^1^ http://solcap.msu.edu/potato_infinium.shtml
